# Sex-specific bacterial microbiome variation in octopus vulgaris skin

**DOI:** 10.3389/fmicb.2023.1233661

**Published:** 2024-01-22

**Authors:** Deiene Rodríguez-Barreto, Juan Carlos Sanz-González, M. Virginia Martín, Jesús M. Arrieta, Eduardo Almansa

**Affiliations:** ^1^Canary Islands Oceanographic Center, Spanish Institute of Oceanography (IEO-CSIC), Santa Cruz de Tenerife, Spain; ^2^University of La Laguna, Santa Cruz de Tenerife, Spain

**Keywords:** cephalopods, octopus, microbiome, skin, sex, aquaculture

## Abstract

Growing knowledge of the host-microbiota of vertebrates has shown the prevalence of sex-specific differences in the microbiome. However, there are virtually no studies assessing sex-associated variation in the microbiome of cephalopods. Here we assess sex-specific variation in the common octopus (*Octopus vulgaris*) skin microbiome using amplicon sequencing targeting the V4 hypervariable region of prokaryote 16S rRNA genes. Skin and mantle-associated mucus was collected from wild adult individuals of common Octopus (*Octopus vulgaris*) (9 males and 7 females of similar size). There were no significant differences in the alpha diversity of microbial communities associated with skin or mantle mucosa between sexes. However, our results clearly indicate that adult octopus males and females have a distinct microbial community composition in both skin and mantle associated mucus communities, with female microbiome being dominated by Firmicutes (48.1%), while that of males contained a majority of Proteobacteria (60.5%), with Firmicutes representing only 3.30%, not finding significant differentiation in the microbial communities between the tissues explored. The dominance of different taxa in the skin of *O. vulgaris* females and males (e.g., Mycoplasmatales and *Lactococcus* in females and Rhizobiales and Rhodobacteriales in males) suggests a sex-specific symbiosis in which those microbes benefit from easy access to distinct substrates present in female and male skin, respectively. Given the lack of differences in size between specimens of both sexes in this study, we hypothesize differences in hormone profile, as well as behavioral or ecological differences between sexes in the wild, as the main drivers of microbiome differentiation between sexes. Most knowledge of cephalopod microbiota is limited to the digestive tract and the reproductive system. However, cephalopod skin is an organ with a plethora of functions. This is a first attempt to characterize cephalopod skin microbiota and determine sex influence on it.

## Highlights

• The dermal microbiota of *Octopus vulgaris* was characterized in adult animals of different sex.
• The dermal microbiome of *O. vulgaris* is considerably more diverse than that described for other tissues.
• Adult male and female octopuses have a significantly different dermal microbial community composition.
• Sex differences must be considered when assessing responses to probiotics/prebiotics or any other treatment in aquaculture research, as their effect may differ between males and females.

## 1 Introduction

Sexual dimorphism can be defined as a sex difference in the mean phenotypic value of traits between females and males in organisms with two sexes (Kaufmann et al., 2021). Those differences can be subtle and can lead to bias between sexes. Gender bias and sexual dimorphism have always been understood as a host-intrinsic factor. However, recent studies suggest that gender bias may be exerted or reinforced by host microbiota, with some sex-specific differences in gene expression and metabolism being driven by sex-specific differences in the microbiota (Flak et al., 2013; Markle et al., 2013; Morris, 2019).

Growing knowledge of the host-microbiota of humans, rodents and other vertebrate taxa, has shown the prevalence of sexual dimorphism in the microbiome (“microsexome”) (Kim, 2022; Mulak et al., 2022). However, there are few studies focusing on sex-specific microbiome variation in non-vertebrate models, mainly on terrestrial animals, with very few studies targeting aquatic organisms, and virtually none targeting cephalopods (Bates et al., 2022).

Cephalopods are keystone invertebrates in some marine ecosystems, valuable fisheries resources (Xavier et al., 2015, 2018; Vieites et al., 2019; Arkhipkin et al., 2021; Lishchenko et al., 2021; Pita et al., 2021; Sauer et al., 2021) and fantastic models to study nervous system complexity and evolution (Albertin and Simakov, 2020). Microbiome research in cephalopods, albeit in its infancy, has expanded over the last decade. Most studies on cephalopod microbiome research till date assess interspecific differences, and do not account for intraspecific differences in sex (Chalifour and Li, 2021; Kang et al., 2022). However, the microbiome of cephalopods is likely to vary in a sex-dependent manner, considering that both sexes often differ in their body mass index and hormone/pheromone production, while displaying dietary, behavioral, and ecological differences that may result in different niches supporting specific microorganisms (Kang et al., 2022).

Most studies on cephalopod microbiome have focused on the microbial diversity of the gut (Iehata et al., 2015; Roura et al., 2017; Cruz-Leyva et al., 2019; Lutz et al., 2019; Kang et al., 2022), and the reproductive tract (Barbieri et al., 2001; Epel, 2001; Collins et al., 2012; Kerwin and Nyholm, 2017). However, the skin microbiome of cephalopods has been largely neglected, with only Lutz et al. (2019) succinctly describing common cuttlefish (*Sepia officinalis*) skin microbiome. Cephalopod skin presents remarkable features, such as light sensitivity (Kingston et al., 2015; Knight, 2015) and reflectance (Mäthger and Hanlon, 2007; Deravi, 2021) that contribute to their outstanding camouflage and signaling capabilities. Cephalopod skin lacks placode keratin derived structures such as hair, feathers, or scales, and expands to the syphon and pallial cavity comprising a huge surface of the cephalopod body (Packard, 1988; Anadón, 2019). Besides its protective function, and its role in camouflage and communication, the skin is involved in a wider variety of functions, including lubrication, osmotic regulation, nutrient and oxygen exchange, or even prey attraction (Packard, 1988; Accogli et al., 2017; Anadón, 2019; González-Costa et al., 2020). Functional microbiome plays an essential role in the host’s health and homeostasis, being involved in a plethora of functions in aquatic organisms, including immunity and protection against opportunistic pathogens (Dawood et al., 2019; Evariste et al., 2019; Yu et al., 2021).

Among cephalopods, the common octopus (*Octopus vulgaris*) is a well-studied and cited model species in neurobiology, ecology, and aquaculture research (Vidal et al., 2014; Albertin and Simakov, 2020; Arkhipkin et al., 2021), which makes it a great candidate to assess intraspecific skin microbiome variations, including those related to sex. Addressing the previously overlooked sex-differences is critically important given that host responses to environmental cues or experimental treatments may differ between sexes, introducing a confounding factor for interpreting research outputs. Thus, considering the plausible relevance of skin microbiome on skin health and function, this study aims to characterize cephalopod skin microbiota and test for the hypothesized sex-related compositional differences. We will do so by describing the skin microbiome (external body surface and mantle cavity) of common octopus (*O. vulgaris*) in wild adult male and female individuals collected on the northwest coast of Tenerife.

## 2 Materials and methods

### 2.1 Sampling

Sixteen wild adult individuals [9 males and 7 females of *Octopus vulgaris* (common octopus)] were collected by professional artisanal fishermen in the northwest coast of Tenerife, Canary Islands Spain (28°30′N, 16°12′W). Upon arrival at the culture facilities of the Centro Oceanográfico de Canarias, sex and weight were determined. A Salter Brecknell 235 Series mechanical hanging scales SALTER MODEL 235 6S with accurate weighing was used (*d* = 100 g; Max = 25 kg). Actual capture dates and individual weights are provided in Table 1. Octopuses were housed individually for acclimatation, anaesthetized by immersion in 2% ethanol till ventilation frequency, depth and coordination all become suppressed, then animals were weighted and either skin-associated mucus or mantle-associated mucus was collected. Skin-associated mucus was collected from each individual by swabbing back and forth along the entire length and surface of the head and body up to 10 times using sterile swabs (Deltalab Code 300265: CE MDD Class I Sterile). Likewise, mantle-associated mucus was collected by swabbing back and forth along the entire length of the mantle cavity walls (MCW), avoiding the contact with gills or any other organ suspended in the inner face of the mantle cavity. All samples were immediately stored at –80°C until DNA extraction.

**TABLE 1:** Sample information summary.

Sex	Tag number	Sampling date	Wet weight (g)
Female	726	12/02/2020	1,150
Female	305	12/02/2020	2,700
Female	2,619	10/03/2020	1,300
Female	1,175	10/03/2020	1,400
Female	996	10/03/2020	1,600
Female	2,310	10/03/2020	1,600
Female	2,658	11/01/2021	1,470
Male	1,635	12/02/2020	1,200
Male	2,410	12/02/2020	1,700
Male	2,619	12/02/2020	1,800
Male	1,568	12/02/2020	1,900
Male	2,934	12/02/2020	2,200
Male	4,450	12/02/2020	2,300
Male	4,346	12/02/2020	2,400
Male	481	11/01/2021	1,250
Male	2,212	11/01/2021	1,420

All animal experiments were performed according to the Spanish law RD53/2013 within the framework of the European Union directive on animal welfare (Directive 2010/63/EU) for the protection of animals employed for experimentation and other scientific purposes, following the Guidelines for the care and welfare of cephalopods published by Fiorito et al. (2015), and approved by the Ethic Committee of the National Competent Authority (project number: CEIBA 2017-0249).

Differences in weight between sexes were assessed with a Wilcoxon rank-sum test.

### 2.2 16S rRNA amplicon sequencing

DNA was extracted using a standard phenol-chloroform protocol (Green and Sambrook, 2017) with slight modifications. We incubated the swabs for 2 h in Lysis buffer (10 mM Tris–HCl, 1 mM EDTA pH 8.3) and proteinase K at 56°C with continuous shaking at 1600 rpm in a thermo-block mixer, followed by phenol-chloroform extraction and ethanol precipitation with sodium acetate.

16S library preparation was performed following a two PCR step preparation protocol for amplification and subsequent index addition (Illumina, 2013). Briefly, we used the primers targeting V4 hypervariable region of the 16S rRNA gene (in bold) with the addition of adapter sequences for dual-index barcodes (over-hangers in italics) for 1st Stage PCR [515F–*A CACTGACGACATGGTTCTACA***GTGYCAGCMGCCGCGGTAA** (Parada et al., 2016); 806R–*TACGGTAGCAGAGACTTGGTCTT***GG** **ACTACNVGGGTWTCTAAT** (Apprill et al., 2015)]. All PCRs were performed in two replicates using 20 μl volume reactions. A total of 2 μl DNA extract or nuclease-free water (negative PCR control) was added to a PCR mixture containing 0.05 μM primers, 0.1 μl methylated Bovine Serum Albumin (mBSA) at 10% (Sigma), 10 μl of Accustart™ II PCR Hot Start Supermix 2X (Quantabio) and diluted with nuclease-free water to a 1X final concentration. Thermal cycling included an initial denaturation at 95°C for 5 min; 35 cycles of denaturation for 30 s at 94°C, annealing for 1 min at 50°C, elongation for 1 min 30 s at 72°C; final elongation at 72°C for 10 min; and storage at 10°C. The two replicates of each reaction were pooled and visualized on TAE 1.8% agarose gel. Product size visualized in the gel was around 380 bp. AMPure XP beads (Beckman Coulter, Brea, CA, USA) were used to purify the 16S V4 amplicon prior to indexing. Dual indices and Illumina sequencing adapters were attached using the Nextera XT Index kit. Thermal cycling for indexing included 12 cycles, with denaturation, annealing and elongation equivalent to the 1St step PCR reaction. Total fragment length after indexing was ∼420 bp. We used AMPure XP beads to clean up the final library before quantification. DNA concentrations were measured with Qubit™ 1X dsDNA HS Assay Kit using Qubit 3 fluorometer (Invitrogen). Equimolar volumes of each PCR product were mixed to a final concentration of 5 nM to create a composite sample for high-throughput sequencing and submitted to MACROGEN sequencing services (Macrogen Spain Inc., Spain; Madrid). The 16S library was denatured and diluted to a final concentration of 1.8 pM, and 30% PhiX control library was used for sequencing. Sequences were obtained using an Illumina MiSeq (San Diego, CA, USA) v3 paired-end 300 bp protocol for 600 cycles.

### 2.3 Microbiome data analysis and statistics

Raw DNA sequence reads were processed using DADA2 (Callahan et al., 2016). Briefly, all reads were, screened for quality, after adaptor removal, the first 20 bp of forward and reverse reads were trimmed to eliminate primers, and truncated based on overall quality score, truncating reads at the first instance of a quality score below 20. Absence of primer sequence and overall quality of sequences were checked after trimming. Reads were then denoised, merged, subject to chimera screening and removal, and non-chimeric sequences were assigned into actual sequence variants (ASVs). Track of reads left after each processing step from raw reads to non-chimeric reads is provided in Supplementary Figure 1. Taxonomic classification of ASVs was performed within DADA using the Silva reference taxonomy (v138.1) with a custom trained classifier (Bokulich et al., 2018). ASVs were further filtered. First, ambiguous, and unwanted taxa at Kingdom and Phylum level (e.g., Eukaryotic and non-identified sequences) were removed, and the remaining ASVs were further filtered based on prevalence removing those ASVs contributing less than 1% of the reads detected for all samples, as well as those phyla appearing in just 1% of the samples, which resulted in the exclusion of “*Cloacimonadota*,”“*Deinococcota*,” and “*Iainarchaeota.*”

Data analyses were performed in R (version 4.1.3). Random subsampling was used to rarefy individual samples to even depth for further downstream analyses using phyloseq version 1.36.0; “set.seed (123)” was used to initialize repeatable random subsampling using rarefy_even_depth phyloseq function (1130 ASVs were removed because they were no longer present in any sample after random subsampling). Only samples that passed the quality filtering and subsampling were used for further analysis. The alpha diversity and Bray–Curtis dissimilarity index (Whittaker, 1972) were also calculated using phyloseq version 1.36.0 (McMurdie and Holmes, 2013). Alpha-diversity indices were calculated at ASV level. Differences in alpha diversity [Observed richness, Chao1 richness (Chao, 1984) and Shannon diversity (Shannon, 1948) between females and males were tested with a Welch two samples *t*-test after checking for normality (visually and statistically (shapiro.test)) and homogeneity of the variance (Bartlett’s test)]. Microbiome structure (beta diversity), based on Bray-Curtis distance, was visualized using non-metric multidimensional scaling (NMDS) ordination. Centered log-ratio (clr) transformation was used as input for multivariate hypothesis testing. Significant differences between groups were determined using a permutational multivariate analysis of variance (PERMANOVA) with 9999 permutations and a *p* < 0.05 cutoff, and a permutation test for multivariate dispersion (PERMDISP) was conducted to test for differences in variance (dispersion) among community samples using vegan package version 2.5-7 (Oksanen et al., 2019). Relative abundances of ASVs were calculated and visualized using the Phyloseq package. Indicator species analysis was performed using the indicspecies package (De Cáceres and Legendre, 2009; De Cáceres et al., 2012) using the “r.g” function and 9999 permutations. Statistical analysis of ASV abundance at different taxonomic levels was performed using DESeq2 (Love et al., 2014). Within the DesSeq2 models, independent filtering of low coverage ASVs was applied, optimizing power for identification of differentially abundant ASVs at a threshold of α = 0.05. Default settings were applied for outlier detection and moderation of ASV level dispersion estimates. ASV abundance was considered significantly different at FDR < 0.05. All Figures were plotted using ggplot2 v.3.3.5 (Wickham, 2009).

## 3 Results

No significant differences in mean weight between females and males were found in this study (*W* = 22, *p*-value = 0.34). Females had a mean wet weight of 1.60 ± 0.51 kg (mean ± sd) while males weighted on average 1.80 ± 0.44 kg. Sample information summary is provided in Table 1.

After processing raw reads and filtering out ambiguous and low prevalence phylum, over half a million reads were retained (599878), with 20045 being the median number of reads per sample. The min. number of reads per sample was 5433 and the max. number of reads was 53384, with no significant differences in sequencing depth between samples belonging to females (21201 ± 12710) and males (24996 ± 11568) (*t* = −0.79089, df = 22.547, *p*-value = 0.4372). The total number of ASVs corresponding to unique sequences was 3020. After subsampling to even depth, 1130 ASVs were removed because they were no longer present in any sample.

**FIGURE 1:**
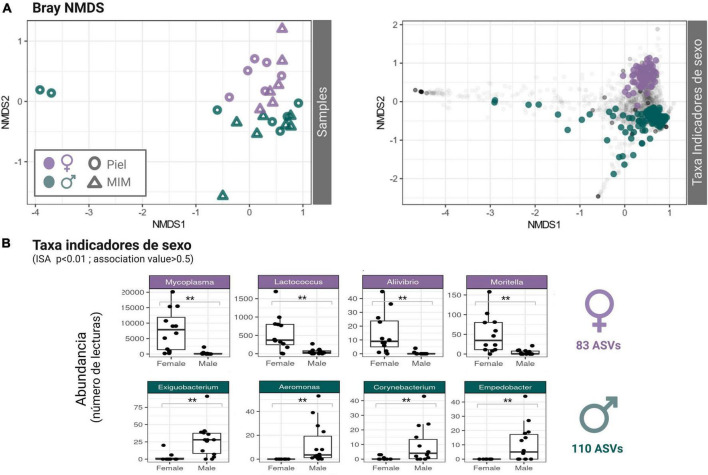
**(A)** Non-metric Multidimensional Scaling (NMDS): Bray-Curtis distance-based community structure [Left: Samples (sample scores); Right: Taxa (represented in color the ASVs identified as sex markers according to Indicator Species Analysis (ISA) = “ISA significant taxa”)]. The NMDS clearly shows that ASVs identified as markers of sex are responsible for the differentiation between males and females. **(B)** Box-and-whisker plot representing the read count distribution (Abundance) of the ASVs identified as sex markers clustered at genus level. For illustrative purposes, only the taxa that presented a significance level **p > 0.01 and an association value greater than 0.5 are represented. A total of 83 ASVs were significantly associated to Females and 110 to Males, the full list is provided in Supplementary Table 1.

**FIGURE 2:**
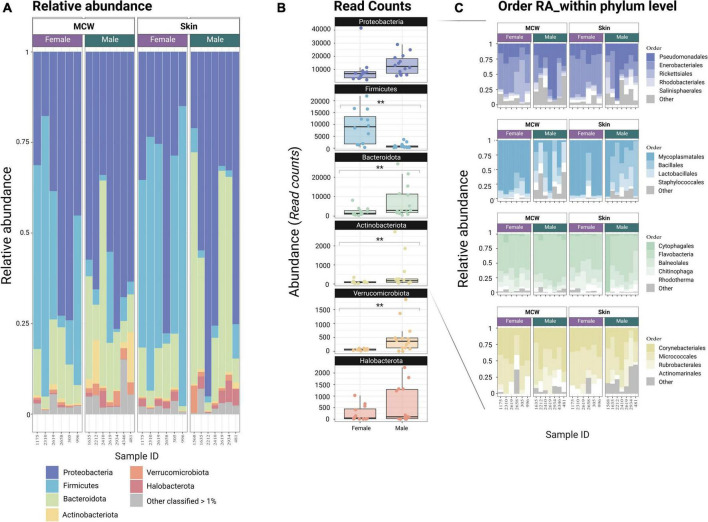
**(A)** Stacked Bar chart representing the relative abundance (RA) at phylum level. For visualization purposes, those phyla with a RA below than 1% were grouped in the category “Others <1%”. **(B)** Box-and-whisker plot representing the read count distribution (Abundance) of the most abundant phyla (RA > 1%) between sexes. **(C)** Stacked Bar chart representing the relative abundance (RA) of the different orders within each Phylum. For visualization purposes, those orders with a RA within phylum below than 1% were grouped in the category “Others” *MCW*: Mantle Cavity Wall. ** Indicates *p* ≤ 0.01.

No significant differences in microbial ASV richness or diversity were detected between *Octopus* females and males in skin neither in mantle mucosa (*p* > 0.05) (*Skin*: Chao1: *t* = −1.24, df = 10.879, *p* = 0.2402; Shannon: *t* = −0.53, df = 11, *p* = 0.604; *MCW*: Chao1: *t* = −0.64, df = 10.978, *p*-value = 0.5356; Shannon: *t* = −0.59, df = 10.172, *p*-value = 0.57; Supplementary Figure 2). Although similarly diverse, the dermal microbial communities of male and female *O. vulgaris* displayed clear differences in community structure and overall composition (Figures 1, 2). There was a significant difference in the community structure between females and males for both skin and MCW microbiome, with sex explaining ∼9% of the variance. Yet, no significant differences were found between skin and mantle microbial communities within each sex, and there was no significant interaction when considering both factors [PERMANOVA; Sex *F*_(1,25)_ = 2.35, *p* = 0.001, Tissue *F*_(1,25)_ = 1.30, *p* = 0.120; Interaction *F*_(1,25)_ = 0.90, *p* = 0.68; PERMDISP *p* > 0.05]. Indicator species analysis (ISA) revealed that from 3020 ASV tested, 83 ASVs were significantly associated to females and 110 to males using a 0.05 significance threshold (Supplementary Table 1). The 83 ASVs identified as female indicators belong to 23 known orders, 36 known families and 40 known genera, while the 110 ASVs identified as male indicators belong to 31 known orders, 42 known families and 48 known genera (Further detail on the taxonomic identity of the ASVs identified as sex-indicator species can be found in Supplementary Table 1). Non-metric multidimensional scaling (NMDS) ordination of microbial community structure based on Bray-Curtis distances (Figure 1A) clearly shows that the ASVs identified as Sex markers (ISA) are responsible for the sample clustering and differentiation between males and females. In addition to the ISA, differential abundance analysis was performed at different taxonomic levels using DESeq2. At genus level, differences in the abundance of 34 genera and 14 unclassified genera of known families were found (FDR < 0.1; Supplementary Figure 3). The identity of those is highlighted in Supplementary Figure 1. Among all of them is worth highlighting the higher abundance of *Mycoplasma*, *Lactococcus*, *Candidatus Bacilloplasma*, *Moritella*, and *Halioxenophilus* in females (DESeq2 log2FoldChange ≥ 1.5; FDR ≤ 0.01) which were also identified as female-indicator species with a *p* ≤ 0.01 and an association value greater than 0.5 (Figure 1B). Among male-indicator species it is worth stressing *Exiguobacterium*, *Pseudomonas*, and a couple of unclassified *Rhodobacteraceae* (*p* ≤ 0.01 and an association value of 0.5) which also displayed a significantly higher abundance in males (DESeq2 FDR ≤ 0.1). Indicator species identified by conventional ISA analysis showed good agreement with the differences in abundance detected between females and males using DESEq2, with many sex indicator species showing significant differences in abundance at genus level (FDR > 0.1; Supplementary Figure 3). Differences in abundance at order and phylum level are reported in Supplementary Figure 4 and Supplementary Table 2, respectively. Among the 24 orders that displayed differential abundance, it is worth noting those with FDR ≤ 0.01 which include: Rhizobiales, Sphingobacteriales, Puniceispirillales, Pseudomonadales, Sphingomonadales, Caulobacterales, Verrucomicrobiales, Corynebacteriales, Flavobacteriales, Rhodobacterales, Enterobacterales, Lactobacillales, and Mycoplasmatales. Several orders belonging to Actinobacteriota and Chloroflexi also displayed differences in abundance (FDR ≤ 0.05).

At the phylum level, the female skin and MCW microbiome was dominated by Firmicutes (median = 48.1%), followed by Proteobacteria (median = 33.2%), and Bacteroidota (13.1%) which jointly account for (∼95%) of the female dermal microbiome. On the other hand, Proteobacteria were by far the most abundant bacterial phylum present in males (median = 60.5%), followed by Bacteroidota (median = 16.9%) and with lower levels of Firmicutes (median = 3.30%), showing significant differences in abundance for some of the most prominent phyla (Supplementary Table 2 and Figures 2A, B). Not only males and females differ at phylum level, but they also do in their compositional profile at lower taxonomic ranks. Thus, among Firmicutes females are dominated by Mycoplasmatales and Lactobacillales, while males are dominated by Bacillales with a wider range of rare taxa (<1%) including Clostridiales, Exiguobacterales, Peptostreptococcales-Tissierellales which are practically not present in females (Figure 2C and Supplementary Figure 4). Within the phylum Proteobacteria, Enterobacteriales are the most prominent order in females (median 54.8%) while Pseudomonadales is the dominant order among males (median = 31%), again displaying a wider variety of taxa at low abundances (Figure 2C and Supplementary Figure 4). Amongst Bacteroidota, Bacteroidales (<1%) are more prevalent in females and Flavobacteriales are more prevalent in males, and among Actinobacteriota is worth noting the lower prevalence of Rubrobacterales in females compared to males (Supplementary Figure 4).

## 4 Discussion

Studies focusing on the sex-related differences in the microbiome of aquatic animals have been reported across different aquatic taxa, mainly targeting vertebrates (Reviewed Bates et al., 2022). The few available studies focusing on invertebrates include corals (Wessels et al., 2017), crustaceans (Wenzel et al., 2018; Clarke et al., 2019), or mollusks (Iehata et al., 2015; Takacs-Vesbach et al., 2016). Yet information on sex-related microbiome differentiation in cephalopods is very scarce, with the only study available in this regard (Iehata et al., 2015), focusing on the bacterial community associated to the digestive tract of wild Chilean Octopus (*Octopus mimus*, Gould 1852) using plate cultures and 16S rDNA clone libraries. Authors revealed bacterial community structure and nutritional enzyme activity differences between sexes, with a higher frequency of Firmicutes isolated in females with respect to males, thus uncovering microbial community functional differences associated to host sex.

We assessed the variation in mucus (skin and MCW) microbial composition of *O. vulgaris*, revealing the presence of sexual differences in microbial community composition. To our knowledge, there is no prior report on the relationship between skin bacterial community and host sex, either in *Octopus* or in any other cephalopods. The fact that their microbiome varies in a sex-dependent manner could be associated to different factors.

### 4.1 Plausible sources of variation

Some authors hypothesize that sexual differences in microbial communities could be linked to sexual size dimorphism or differences in body mass between the specimens studied within each sex (Veuille, 1980; Gao et al., 2018), however, the lack of significant differences in size or weight between males and females in this study suggests that is not the case here. The fact that both sexes were captured and sampled within the same timeframe (season) and within the same capture zone allows us to discard co-founding seasonal and regional variation as well. We acknowledge that for both sexes we have samples from 2 different years (same season), which allow us to account for the natural individual variation that may occur between cohorts. Sex-specific differences in habitat selection could play a role in the sex differences observed in Octopus skin, since they are likely to display dietary, behavioral, and ecological differences that support niche-specific microorganisms. The scarcity of field, or experimental studies purposely assessing differences between sexes, highlights the need to account for sex differences in ecological/biological studies, which not only will contribute to understand the complex dynamics of this species in the wild, but also its influence on Octopus microbiome, allowing to further validate our hypotheses.

Iehata et al. (2015) hypothesized differences in feeding habits between females and males as the most plausible player in both, enzymatic activity and bacterial community differences between sexes in the gut microbiome of Chilean octopus. On the other hand, in terrestrial vertebrates it has been demonstrated that dietary selection and feeding strategies from different ecological niches chosen by different sexes can significantly influence the composition of gut microbiota (Zhu et al., 2020), with studies in fish also showing that diet and feeding habits strongly influence gut microbiota (Wei et al., 2018; Xu et al., 2019). Thus, differences in feeding habits could also be partially responsible for the differences between sexes detected in *O. vulgaris* skin microbiome. Although, the skin microbiota of cephalopods or even farmed fish species and its response to diet remains largely unmapped, the few studies available on fish reveal that skin microbiome is dependent on their diet and the environmental conditions fish are exposed to Uren Webster et al. (2020). In this regard, recent studies in humans and mammal models (De Pessemier et al., 2021) indicate a close relationship between the skin and gut microbiome, although the underlying mechanisms are poorly understood.

Besides food, differences in niche preferences between males and females could also support niche-specific microorganisms that may explain to some extent the differences in microbial composition observed between sexes in this study. However, despite the growing knowledge of *O. vulgaris* ecology, sex-specific differences in niche preferences have not been reported for this species. The fact that *O. vulgaris* is a species that can be found in a wide range of habitats, from shallow waters up to 100 m depth, in a wide range of substrates (Lishchenko et al., 2021; Sauer et al., 2021) and the difficulty of assessing their sex non-invasively in the wild, make really difficult to actually evaluate whether there are sex-specific differences in niche preferences. Tofeil and Dridi (2022), in a study that span over 10 years on the coast of Morocco, revealed a significant variation in female space occupation and depth distribution between two different areas, with important seasonal fluctuations (Tofeil and Dridi, 2022), but no information was reported about male distribution. Sheltering behavior (natural dens and artificial nest preference) and habitat preferences (sea-grass vs. sandy bottom) of common Octopus were assessed by Ulaş et al. (2019), in the Aegean Sea. Their results reveal no significant sex effect in either of the parameters considered, nonetheless if the data would have been statistically analyzed considering sex preference by stratum depth, the conclusions reached may have changed. Thus, sex differences in niche preferences cannot be confirmed neither rejected as further studies assessing sex-specific niche preferences are needed to understand the complex dynamics of this species in the wild and its influence on Octopus microbiome, which could ultimately affect important aspects such a: metabolism, immunity or the behavior of this species.

Differences in hormone production and neuroendocrine profile between females and males could also mediate changes in the microbial community profile, both directly and indirectly, through modulation of behavior. There is a high differentiation in signaling pathways when comparing female and male transcriptomic profiles of White bodies (optic lobes) in *Octopus maya*, with, for instance, androgen receptor-signaling pathway being detected only in males, whereas estrogen receptor showing higher expression in females. This and other studies suggest steroid hormones are involved in female and male physiological dimorphism during reproduction (Juárez et al., 2019; Di Cristo, 2021). In vertebrates, sex-specific differences in skin microbial communities have been associated with changes in circulating sex hormones (Shin et al., 2019; Park et al., 2022). But this association is bidirectional, since hormonal profile can also be affected/modulated by changes in the microbiome (Weger et al., 2019). Sex hormones can affect immune cell functioning, as well as behavior, modulating microbial communities and being at the same time modulated by different microbes, resulting in a complex bidirectional interaction (Kim and Benayoun, 2020; Skowron et al., 2021; Kim et al., 2022). Hence, we suggest that differences in neuroendocrine profiles between both sexes are likely linked to the differences in skin microbial composition found in the present study.

Sex-related variability in skin features such as skin pH, thickness, mucus secretion and composition could also be responsible for the differences in the microbial community observed. The physical and chemical properties of the skin influence the dominance of specific microbiota, their proportions, and their mutual relationships (Skowron et al., 2021). However, those are features uncharacterized in cephalopods. Cephalopod skin is involved in a wide variety of functions, including camouflage, communication, and osmoregulation, among others. These skin functions may be affected by the skin microbiome, as it has been found for other organs where the microbiota plays an essential role in the host health and homeostasis. Our findings highlight the need to further explore the skin microbiome of cephalopods, to better understand the origin of the sex-specific differences observed here, and to determine whether those differences are ubiquitously found and have further consequences in cephalopods in general, and in *O. vulgaris* ecology and behavior in particular.

In addition to all the plausible factors discussed above that could affect Octopus skin microbiome in a sex -dependent manner, we would like to draw attention to the fact that both sexes of this species have completely different life strategies. O. *vulgaris* females cease feeding after laying their eggs and focus only on their care until the hatching of the paralarvae, while males do not look after their offspring (Vidal et al., 2014). Although all females used in this study were collected before the brooding period, there is the possibility that the differences in the microbial community between sexes could be also linked to some extent to physiological changes related to the pre-spawning period. This is not a far-fetched idea considering that the optic lobe transcriptome of *O. maya* (Juárez et al., 2019) shows an enrichment in genes associated to starvation in pre-spawning females, suggesting that females experience subtle changes in their neuroendocrine pathways even before the anorexic behavior is observed in fertilized females.

### 4.2 Sex specific differences: taxa and implications

Focusing on the specific differences detected in our study, the most striking one is the higher abundance of Firmicutes, Mycoplasmatales and Lactobacillales in particular, in females. These results are consistent to those described by Iehata et al. (2015) for *Octopus mimus* gut microbiome, where the authors found differences in the bacterial community composition (culturable and non-culturable) between female and male samples, with a higher prevalence of culturable Firmicutes in Females and a dominance of *Mycoplasma* among the non-culturable taxa, as observed in *O. vulgaris* skin microbiome in this study. *Mycoplasma* has been identified as a core taxon in cephalopods, being the most prevalent taxonomic group in *O. vulgaris* and *O. mimus* gut microbiome (Iehata et al., 2015; Kang et al., 2022). While some *Mycoplasma* species have been reported to be pathogens or parasites (Razin, 2006), *Mycoplasma* are normal inhabitants of the gut of some aquatic animals, including fish and cephalopods (Mora-Sánchez et al., 2020a,b; Kang et al., 2022; Ma et al., 2022). Our results suggest that *Mycoplasma* is also a natural member of the bacterial community of *O. vulgaris* skin mucus. Despite their prevalence in the digestive tract across taxa, the function and role of *Mycoplasma* in the health of fish and cephalopods remain unclear. Rimoldi et al. (2019) suggested that *Mycoplasma* may have a positive impact on farmed rainbow trout health by producing lactic acid and acetic acid. Enhanced health conditions (Bozzi et al., 2021), disease resilience (Rasmussen et al., 2022), and improved growth performance (Rimoldi et al., 2019; Bozzi et al., 2021) have also been associated to *Mycoplasma* sp. in salmonids. The beneficial role of *Mycoplasma* sp. is further supported by the fact that reduced abundance of *Mycoplasma* sp. coincides with the increased prevalence of pathogenic/opportunistic bacteria in the digestive tract of salmonids (Scheuring et al., 2022). That could be the case also in skin, however, such hypothesis needs validation. Therefore, further investigation on the role of *Mycoplasma* on skin and intestinal tract of fish and cephalopods is needed to better understand the differences between sexes.

Within the genus Lactococcus, which also shows a significantly higher prevalence in females than in males in this study, we can find opportunistic pathogens such as *Lactococcus garvieae*, etiological agent of Lactococcosis in fish (Vendrell et al., 2006), and beneficial bacteria such as *Lactococcus lactis*, used as probiotics in fish aquaculture due to its ability to promote host health by reducing pathogenic bacteria, increasing food nutritional value, and enhancing the host immune response (Cano-Lozano et al., 2021; Pereira et al., 2022).

Although some species of *Mycoplasma* and *Lactococcus* have pathogenic potential, members of these genera appear to be autochthonous inhabitants of apparently healthy cephalopods. suggesting that they are non-pathogenic for the host, and that the differences between females and males are rather associated to the extrinsic and intrinsic factors previously discussed in this manuscript. As some members of these genera produce lactic acid or acetic acid as their major metabolites (Holben et al., 2002; König and Fröhlich, 2017), the dominance of these taxa in *O. vulgaris* female skin could suggest a sex-specific symbiosis in which these microbes benefit from easy access to fermentable substrates present in female skin that may not be present in the mucus of males. However, these are speculations since the research on the functional role of symbiotic and commensal bacteria in *Octopus* microbiome is still in its infancy.

Another genus that was differentially distributed between males and females is *Candidatus* Bacilloplasma, which is considered a novel lineage of class Mollicutes (Kostanjsek et al., 2007). This species is the predominant taxa or one of the major genera in the digestive tract of many Crustacea [e.g., crayfish, *Procambarus clarkia*, (Shui et al., 2020; Feng et al., 2021), woodlouse, *Porcellio scaber* (Kostanjsek et al., 2007), white shrimp, *Penaeus vannamei* (Hou et al., 2018; Wang et al., 2020)]. The role of *Candidatus* Bacilloplasma is unclear, thus it is difficult to speculate about the potential role of this rod-shaped mollicutes in Octopus skin. As crustaceans are part of the natural diet of *O. vulgaris* (Lishchenko et al., 2021), we hypothesize that differences in the prevalence of these taxa between females and males could support and be related to differences in feeding habits between sexes.

*Gammaproteobacteria* belonging to the genus *Halioxenophilus*, *Aliivibrio*, *Moritella* were also indicator taxa for females. *Halioxenophilus* is a recently discovered genus isolated from seawater, known to degrade xylene (Iwaki et al., 2018). This taxon has been found as part of the calcareous sponge *Sycettusa hastifera* microbiome, being responsive to thermal and pH stress reducing its relative abundance (Ribeiro et al., 2021). Yet, there is little we can say about the presence and role of this marine bacteria in Octopus female skin.

The bioluminescent *bacterium Aliivibrio fischeri* has been reported as the only microorganism in the light organs of bob-tail squids, *Euprymna scolopes*, in a single microorganism symbiotic host-bacteria association (Nyholm and McFall-Ngai, 2004). *Aliivibrio* is also relatively abundant in Beka squid (*Loliolus beka*), being part of the core microbiome of this cephalopod (Kang et al., 2022). However, *Aliivibrio* sp. is also commonly found in the gastrointestinal tract of fish (Burtseva et al., 2021), as a pathogenic/opportunistic bacterium becoming more abundant or even dominant in stressed fish (Godoy et al., 2015; Scheuring et al., 2022). These pathogenic strains may coexist with other mutualist bacteria such as *Mycoplasma* being occasionally observed at low abundances in healthy individuals (Scheuring et al., 2022). In the current study, *Aliivibrio* is a rare taxon with really low abundance in females, being a female indicator species despite that, because it is virtually absent in male individuals. Thus, its low presence and coexistence with *Mycoplasma* in female skin suggest an anecdotic non-pathogenic presence in Octopus female skin.

The genus *Moritella*, as observed for other Gammaproteobacteria such *Aliivibrio*, was detected in really low abundances in females, and virtually absent in males. Members of this genus are generally psychrophilic and are often associated to deep-sea water, deep-sea organisms and ocean sediments (Nichols, 2003; Urakawa, 2014). Most of those species are not pathogenic, except for *Moritella viscosa*, being the only species so far associated to fish pathogenicity, causing winter-ulcer disease in farmed salmonids (Benediktsdóttir et al., 2000; Karlsen et al., 2017).

*Exiguobacterium*, was identified as a male indicator species, detected in really low abundances in males, and virtually absent in females. Members of Exiguobacterium isolated from different sources, have been associated to the production of cellulases, amylases, xylanases, and ligninases (González-Escobar et al., 2020) as well as the production of the shell component chondroitin (Bhotmange and Singhal, 2015), and have been shown to have potential probiotic functions (Cong et al., 2017). Different strains have been used as probiotics in several invertebrate species, including the shrimp, *P. vannamei*, improving their growth and survival (Sombatjinda et al., 2014; Kim et al., 2022) or the abalone *Haliotis iris* (Tuterangiwhiu, 2015; Grandiosa et al., 2018).

Several Rhizobiales, Rhodobacterales, among other groups, were within the list of male indicator taxa, being significantly more abundant in the skin of male Octopuses. Rhizobiales are quite common nitrogen fixing rhizobial symbionts of legumes. Lipo-chitin oligosaccharides (LCOs) are key signal molecules for nodule development key in the initial stages of *Rhizobium*-legume symbiosis, still another kind of symbiosis between rhizobia may be possible (Chambon et al., 2015). β-chitin is found in the epidermis and the eyes of cephalopods (i.e., in the iridophores) with a content varying from 20 to 40%, and it can be used as a substrate for LCOs production by rhizobia (Berezina, 2016). Therefore, its presence in Octopus skin is not surprising, and suggests that they may be involved in Rhizobium- iridophores symbiosis that may be relevant for light reflectance, and thus Octopus communication, which should be further explored. The differences in the prevalence of Rhizobiales between females and males may be related and support sex-specific differences in skin between both sexes.

Alpha-proteobacteria including Rhodobacterales, Rhizobiales are symbiotic bacteria quite prevalent in nidamental gland and egg sheath of several squid and cuttlefish species including the bobtail squid, *Euprymna scolopes*, (Collins et al., 2012; Kerwin and Nyholm, 2017), the arrow squid, *Loligo pealei* (Barbieri et al., 2001), the common cuttlefish, *Sepia officinalis*, and the pharaoh cuttlefish, *Sepia pharaonis* (Epel, 2001). Members of Rhodobacterales can also be found free-living in a relatively high abundance in the water column in their natural habitat (Collins et al., 2015; Kerwin and Nyholm, 2017). Rhodobacterales found in the reproductive system of squid frequently generate pigments, being likely linked to the coloration of the nidamental gland of squid and cuttlefish (Collins et al., 2012; Kerwin and Nyholm, 2017). We hypothesize that the sex-specific differences observed in *O. vulgaris* skin could be related to differences in habitat preferences between sexes and/or skin pigmentation variations between males and females.

### 4.3 Relevance of accounting for sex differences in octopus research

The results obtained from this study are especially relevant for aquaculture. The use of probiotics and prebiotics has become a cornerstone in aquaculture research (Akhter et al., 2015; Hai, 2015; Ringø, 2020). However, given the differences observed in this study, the efficacy of these treatments can vary between sexes. This has been shown in mammals (Shastri et al., 2015; Lee et al., 2017; Christoforidou et al., 2019; Murray et al., 2019). Likewise, species can respond to dietary treatments or any other experimental treatment in a sex-dependent manner (Bolnick et al., 2014; Baars et al., 2018; Navarro-Barrón et al., 2019; Peng et al., 2020; Xu et al., 2022). Yet, few studies have taken sex into account in aquaculture research (Bates et al., 2022). Addressing this oversight is critically important given that optimal host responses to probiotic/prebiotics or any other treatment can differ between sexes (Bates et al., 2022). Here we have shown that *O. vulgaris* skin microbiome varies in a sex-dependent manner, a fact that has been overlooked in prior studies. Since *Octopus* is a model species in neurobiology, ecology, and aquaculture research, our study stresses the importance of accounting for sex differences when assessing host responses to environmental cues or experimental treatments to avoid the confounding effect of sex-related responses.

## 5 Conclusion

In this study we reveal the presence of sex differences in the dermic microbial community composition of O. vulgaris. From this study, we can conclude that *O. vulgaris* skin microbiome is considerably more diverse than that described for other tissues in the literature. Given the lack of differences in body weight between sexes in this study, we hypothesize that sex-specific differences in habitat selection, feeding habits as well as physiological, hormonal, and topographical differences in Octopus skin between males and females are the most likely drivers of the differences in the microbial composition observed. The scarcity of field or experimental studies purposely assessing differences between sexes makes it difficult to find additional support for these findings in previous studies. The dominance of certain distinct taxa in the skin of female and males *O. vulgaris* (such as Mycoplasmatales and *Lactococcus* in females and Rhizobiales and Rhodobacteriales in males) suggests a sex-specific symbiosis in which those microbes benefit from easy access to specific substrates present in the skin of female and male individuals, respectively. This hypothesis needs to be validated, thus further research on potential functional role of symbiotic and commensal bacteria in Octopus microbiome is needed.

Despite intense interest and some research progress, much of our knowledge on the microbiota of *Octopus* and that of other cephalopods is limited to the digestive tract and the reproductive system, however, cephalopod skin is a key organ with multiple functions. This is the first attempt to characterize cephalopod skin microbiota and its association to sex. The admittedly small sample size of our study begs for further studies, including bigger sample sizes and additional Octopus populations of different geographical provenance in order to validate the extent of the observed sex-specific variations.

The overall factors governing the structure of the microbiota (including host sex) are poorly understood.

This study highlights the need to account for sex-specific variability in Octopus microbiome studies specifically and in cephalopod research in general, what will contribute to a more comprehensive interpretation of the research outputs, laying the groundwork to further explore the relationship between skin microbiome and skin functionality in cephalopods.

## Data availability statement

The data presented in the study are deposited in NCBI repository (www.ncbi.nlm.nih.gov), Accession: PRJNA1017903; SAMN37414691-SAMN37414717.

## Ethics statement

The animal study was approved by the Ethic Committee of the National Competent Authority (project number: CEIBA 2017-0249). The study was conducted in accordance with the local legislation and institutional requirements.

## Author contributions

DR-B: conception and design of the work, sample collection and processing (i.e., DNA extraction, library preparation), data analysis and interpretation, drafted the manuscript, critical revision of the article, and final approval of the version to be published. JS-G: sample collection and critical revision of the article and final approval of the version to be published. MM, JA, and EA: conception or design of the work, critical revision of the article, and final approval of the version to be published. All authors contributed to the article and approved the submitted version.
