# Supplementary material for: Sex-specific bacterial microbiome variation in octopus vulgaris skin
Source: Front Microbiol. 2024 Jan 22;14:1233661. doi: 10.3389/fmicb.2023.1233661 (PMC10842966; doi:10.3389/fmicb.2023.1233661)

**Table S1.** **ASVs Indicator species analysis (ISA) summary result table.** Among the 3020 ASV tested, at a significant level of 0.05, 83 ASVs were significantly associated to Females (index 1; color code: purple) and 110 to Males (index 2; color code:green). Stat=association value; sign: * ≤ 0.05; **≤0.01; ***≤0.001.

| **index** | **stat** | **p.value** | **sign.** | **Phylum** | **Class** | **Order** | **Family** | **Genus** |
| --- | --- | --- | --- | --- | --- | --- | --- | --- |
| 1 | 0,49 | 0,0034 | ** | Actinobacteriota | Actinobacteria | Corynebacteriales | Corynebacteriaceae | Corynebacterium |
| 1 | 0,38 | 0,0401 | * | Bacteroidota | Bacteroidia | Bacteroidales | Marinifilaceae | Unclassified_Marinifilaceae |
| 1 | 0,44 | 0,0069 | ** | Bacteroidota | Bacteroidia | Bacteroidales | Prolixibacteraceae | Roseimarinus |
| 1 | 0,37 | 0,0322 | * | Bacteroidota | Bacteroidia | Chitinophagales | Unclassified | Unclassified |
| 1 | 0,23 | 0,0001 | *** | Bacteroidota | Bacteroidia | Chitinophagales | Saprospiraceae | Lewinella |
| 1 | 0,33 | 0,0415 | * | Bacteroidota | Bacteroidia | Chitinophagales | Saprospiraceae | Lewinella |
| 1 | 0,36 | 0,0438 | * | Bacteroidota | Bacteroidia | Chitinophagales | Saprospiraceae | Lewinella |
| 1 | 0,37 | 0,0003 | *** | Bacteroidota | Bacteroidia | Chitinophagales | Saprospiraceae | Lewinella |
| 1 | 0,45 | 0,0135 | * | Bacteroidota | Bacteroidia | Chitinophagales | Saprospiraceae | Lewinella |
| 1 | 0,30 | 0,0481 | * | Bacteroidota | Bacteroidia | Chitinophagales | Saprospiraceae | Membranicola |
| 1 | 0,28 | 0,0021 | ** | Bacteroidota | Bacteroidia | Chitinophagales | Saprospiraceae | Unclassified_Saprospiraceae |
| 1 | 0,33 | 0,0476 | * | Bacteroidota | Bacteroidia | Chitinophagales | Saprospiraceae | Unclassified_Saprospiraceae |
| 1 | 0,34 | 0,0439 | * | Bacteroidota | Bacteroidia | Chitinophagales | Saprospiraceae | Unclassified_Saprospiraceae |
| 1 | 0,36 | 0,0130 | * | Bacteroidota | Bacteroidia | Chitinophagales | Saprospiraceae | Unclassified_Saprospiraceae |
| 1 | 0,27 | 0,0264 | * | Bacteroidota | Bacteroidia | Cytophagales | Cyclobacteriaceae | Cyclobacterium |
| 1 | 0,35 | 0,0233 | * | Bacteroidota | Bacteroidia | Cytophagales | Cyclobacteriaceae | Marivirga |
| 1 | 0,37 | 0,0190 | * | Bacteroidota | Bacteroidia | Flavobacteriales | Crocinitomicaceae | Salinirepens |
| 1 | 0,32 | 0,0446 | * | Bacteroidota | Bacteroidia | Flavobacteriales | Flavobacteriaceae | Arenibacter |
| 1 | 0,39 | 0,0140 | * | Bacteroidota | Bacteroidia | Flavobacteriales | Flavobacteriaceae | Dokdonia |
| 1 | 0,48 | 0,0084 | ** | Bacteroidota | Bacteroidia | Flavobacteriales | Flavobacteriaceae | Dokdonia |
| 1 | 0,33 | 0,0483 | * | Bacteroidota | Bacteroidia | Flavobacteriales | Flavobacteriaceae | MarixanthomoUnclassifieds |
| 1 | 0,44 | 0,0123 | * | Bacteroidota | Bacteroidia | Flavobacteriales | Flavobacteriaceae | MarixanthomoUnclassifieds |
| 1 | 0,35 | 0,0167 | * | Bacteroidota | Bacteroidia | Flavobacteriales | Flavobacteriaceae | Unclassified |
| 1 | 0,42 | 0,0161 | * | Bacteroidota | Bacteroidia | Flavobacteriales | Flavobacteriaceae | Unclassified |
| 1 | 0,44 | 0,0055 | ** | Bacteroidota | Bacteroidia | Flavobacteriales | Flavobacteriaceae | Olleya |
| 1 | 0,63 | 0,0003 | *** | Bacteroidota | Bacteroidia | Flavobacteriales | Flavobacteriaceae | Olleya |
| 1 | 0,32 | 0,0157 | * | Bacteroidota | Bacteroidia | Flavobacteriales | Flavobacteriaceae | Ulvibacter |
| 1 | 0,28 | 0,0424 | * | Bacteroidota | Bacteroidia | Unclassified | Unclassified | Unclassified |
| 1 | 0,33 | 0,0426 | * | Bdellovibrionota | Bdellovibrionia | Bdellovibrionales | Bdellovibrionaceae | OM27 clade |
| 1 | 0,34 | 0,0265 | * | Campylobacterota | Campylobacteria | Campylobacterales | Arcobacteraceae | Unclassified_Arcobacteraceae |
| 1 | 0,26 | 0,0413 | * | Fibrobacterota | Fibrobacteria | Fibrobacterales | Fibrobacteraceae | Unclassified_Fibrobacteraceae |
| 1 | 0,30 | 0,0413 | * | Fibrobacterota | Fibrobacteria | Fibrobacterales | Fibrobacteraceae | Unclassified_Fibrobacteraceae |
| 1 | 0,55 | 0,0004 | *** | Firmicutes | Bacilli | Lactobacillales | Streptococcaceae | Lactococcus |
| 1 | 0,51 | 0,0001 | *** | Firmicutes | Bacilli | Mycoplasmatales | Mycoplasmataceae | Candidatus Bacilloplasma |
| 1 | 0,34 | 0,0030 | ** | Firmicutes | Bacilli | Mycoplasmatales | Mycoplasmataceae | Mycoplasma |
| 1 | 0,35 | 0,0376 | * | Firmicutes | Bacilli | Mycoplasmatales | Mycoplasmataceae | Mycoplasma |
| 1 | 0,44 | 0,0155 | * | Firmicutes | Bacilli | Mycoplasmatales | Mycoplasmataceae | Mycoplasma |
| 1 | 0,45 | 0,0073 | ** | Firmicutes | Bacilli | Mycoplasmatales | Mycoplasmataceae | Mycoplasma |
| 1 | 0,48 | 0,0002 | *** | Firmicutes | Bacilli | Mycoplasmatales | Mycoplasmataceae | Mycoplasma |
| 1 | 0,51 | 0,0001 | *** | Firmicutes | Bacilli | Mycoplasmatales | Mycoplasmataceae | Mycoplasma |
| 1 | 0,53 | 0,0010 | *** | Firmicutes | Bacilli | Mycoplasmatales | Mycoplasmataceae | Mycoplasma |
| 1 | 0,54 | 0,0004 | *** | Firmicutes | Bacilli | Mycoplasmatales | Mycoplasmataceae | Mycoplasma |
| 1 | 0,54 | 0,0008 | *** | Firmicutes | Bacilli | Mycoplasmatales | Mycoplasmataceae | Mycoplasma |
| 1 | 0,38 | 0,0476 | * | Fusobacteriota | Fusobacteriia | Fusobacteriales | Fusobacteriaceae | Psychrilyobacter |
| 1 | 0,39 | 0,0391 | * | Fusobacteriota | Fusobacteriia | Fusobacteriales | Fusobacteriaceae | Psychrilyobacter |
| 1 | 0,30 | 0,0323 | * | Planctomycetota | Planctomycetes | Planctomycetales | Gimesiaceae | Gimesia |
| 1 | 0,38 | 0,0194 | * | Proteobacteria | Alphaproteobacteria | Caulobacterales | Parvularculaceae | Parvularcula |
| 1 | 0,49 | 0,0021 | ** | Proteobacteria | Alphaproteobacteria | Rhodobacterales | Rhodobacteraceae | Litoreibacter |
| 1 | 0,28 | 0,0425 | * | Proteobacteria | Alphaproteobacteria | Rhodobacterales | Rhodobacteraceae | Unclassified |
| 1 | 0,42 | 0,0276 | * | Proteobacteria | Alphaproteobacteria | Rhodobacterales | Rhodobacteraceae | Planktotalea |
| 1 | 0,43 | 0,0158 | * | Proteobacteria | Alphaproteobacteria | Rhodobacterales | Rhodobacteraceae | Planktotalea |
| 1 | 0,38 | 0,0368 | * | Proteobacteria | Alphaproteobacteria | Rhodobacterales | Rhodobacteraceae | Pseudophaeobacter |
| 1 | 0,40 | 0,0435 | * | Proteobacteria | Alphaproteobacteria | Rhodobacterales | Rhodobacteraceae | Pseudophaeobacter |
| 1 | 0,37 | 0,0264 | * | Proteobacteria | Alphaproteobacteria | Rickettsiales | Rickettsiaceae | Unclassified_Rickettsiaceae |
| 1 | 0,38 | 0,0406 | * | Proteobacteria | Alphaproteobacteria | Rickettsiales | Rickettsiaceae | Unclassified_Rickettsiaceae |
| 1 | 0,40 | 0,0417 | * | Proteobacteria | Alphaproteobacteria | SAR11 clade | Clade IV | Unclassified |
| 1 | 0,32 | 0,0163 | * | Proteobacteria | Alphaproteobacteria | Sphingomonadales | Sphingomonadaceae | Erythrobacter |
| 1 | 0,32 | 0,0015 | ** | Proteobacteria | Gammaproteobacteria | Enterobacterales | Colwelliaceae | Colwellia |
| 1 | 0,36 | 0,0096 | ** | Proteobacteria | Gammaproteobacteria | Enterobacterales | Colwelliaceae | Colwellia |
| 1 | 0,42 | 0,0177 | * | Proteobacteria | Gammaproteobacteria | Enterobacterales | Colwelliaceae | Thalassotalea |
| 1 | 0,49 | 0,0086 | ** | Proteobacteria | Gammaproteobacteria | Enterobacterales | Colwelliaceae | Thalassotalea |
| 1 | 0,41 | 0,0067 | ** | Proteobacteria | Gammaproteobacteria | Enterobacterales | Idiomarinaceae | Idiomarina |
| 1 | 0,54 | 0,0002 | *** | Proteobacteria | Gammaproteobacteria | Enterobacterales | Moritellaceae | Moritella |
| 1 | 0,28 | 0,0389 | * | Proteobacteria | Gammaproteobacteria | Enterobacterales | Pasteurellaceae | Haemophilus |
| 1 | 0,56 | 0,0001 | *** | Proteobacteria | Gammaproteobacteria | Enterobacterales | Vibrionaceae | Aliivibrio |
| 1 | 0,34 | 0,0064 | ** | Proteobacteria | Gammaproteobacteria | Enterobacterales | Vibrionaceae | Vibrio |
| 1 | 0,37 | 0,0130 | * | Proteobacteria | Gammaproteobacteria | Enterobacterales | Vibrionaceae | Vibrio |
| 1 | 0,54 | 0,0007 | *** | Proteobacteria | Gammaproteobacteria | Enterobacterales | Vibrionaceae | Vibrio |
| 1 | 0,63 | 0,0002 | *** | Proteobacteria | Gammaproteobacteria | Enterobacterales | Vibrionaceae | Vibrio |
| 1 | 0,31 | 0,0389 | * | Proteobacteria | Gammaproteobacteria | Unclassified_gammproteo | Unclassified_gammproteo | Unclassified_gammproteo |
| 1 | 0,41 | 0,0315 | * | Proteobacteria | Gammaproteobacteria | Pseudomonadales | Alcanivoracaceae1 | Alcanivorax |
| 1 | 0,51 | 0,0022 | ** | Proteobacteria | Gammaproteobacteria | Pseudomonadales | Cellvibrionaceae | Halioxenophilus |
| 1 | 0,34 | 0,0350 | * | Proteobacteria | Gammaproteobacteria | Pseudomonadales | Marinobacteraceae | Marinobacter |
| 1 | 0,39 | 0,0458 | * | Proteobacteria | Gammaproteobacteria | Pseudomonadales | Moraxellaceae | Acinetobacter |
| 1 | 0,31 | 0,0388 | * | Proteobacteria | Gammaproteobacteria | Pseudomonadales | Spongiibacteraceae | Unclassified |
| 1 | 0,30 | 0,0407 | * | Proteobacteria | Gammaproteobacteria | Pseudomonadales | Spongiibacteraceae | Spongiibacter |
| 1 | 0,32 | 0,0031 | ** | Proteobacteria | Gammaproteobacteria | Pseudomonadales | Spongiibacteraceae | Spongiibacter |
| 1 | 0,30 | 0,0131 | * | Proteobacteria | Gammaproteobacteria | Salinisphaerales | Salinisphaeraceae | Salinisphaera |
| 1 | 0,40 | 0,0016 | ** | Proteobacteria | Gammaproteobacteria | Salinisphaerales | Salinisphaeraceae | Salinisphaera |
| 1 | 0,33 | 0,0427 | * | Proteobacteria | Gammaproteobacteria | Salinisphaerales | Solimonadaceae | Oceanococcus |
| 1 | 0,38 | 0,0432 | * | SAR324 clade(Marine group B) | Unclassified_SAR324 clade | Unclassified_SAR324 clade | Unclassified_SAR324 clade | Unclassified_SAR324 clade |
| 1 | 0,47 | 0,0024 | ** | Spirochaetota | Spirochaetia | Spirochaetales | Spirochaetaceae | Unclassified |
| 1 | 0,37 | 0,0395 | * | Verrucomicrobiota | Verrucomicrobiae | Verrucomicrobiales | Rubritaleaceae | Persicirhabdus |
| 2 | 0,31 | 0,0377 | * | Actinobacteriota | Acidimicrobiia | Microtrichales | Microtrichaceae | Unclassified |
| 2 | 0,38 | 0,0489 | * | Actinobacteriota | Acidimicrobiia | Microtrichales | Microtrichaceae | Sva0996 marine group |
| 2 | 0,42 | 0,0067 | ** | Actinobacteriota | Acidimicrobiia | Microtrichales | Microtrichaceae | Sva0996 marine group |
| 2 | 0,43 | 0,0029 | ** | Actinobacteriota | Acidimicrobiia | Microtrichales | Microtrichaceae | Sva0996 marine group |
| 2 | 0,42 | 0,0149 | * | Actinobacteriota | Actinobacteria | Corynebacteriales | Corynebacteriaceae | Corynebacterium |
| 2 | 0,43 | 0,0146 | * | Actinobacteriota | Actinobacteria | Corynebacteriales | Corynebacteriaceae | Corynebacterium |
| 2 | 0,45 | 0,0036 | ** | Actinobacteriota | Actinobacteria | Corynebacteriales | Corynebacteriaceae | Corynebacterium |
| 2 | 0,40 | 0,0150 | * | Actinobacteriota | Rubrobacteria | Rubrobacterales | Rubrobacteriaceae | Rubrobacter |
| 2 | 0,24 | 0,0431 | * | Bacteroidota | Bacteroidia | Chitinophagales | Unclassified_Chitinophagales | Unclassified_Chitinophagales |
| 2 | 0,38 | 0,0389 | * | Bacteroidota | Bacteroidia | Chitinophagales | Saprospiraceae | Lewinella |
| 2 | 0,27 | 0,0433 | * | Bacteroidota | Bacteroidia | Chitinophagales | Saprospiraceae | Unclassified_Saprospiraceae |
| 2 | 0,42 | 0,0390 | * | Bacteroidota | Bacteroidia | Chitinophagales | Saprospiraceae | Unclassified_Saprospiraceae |
| 2 | 0,30 | 0,0263 | * | Bacteroidota | Bacteroidia | Chitinophagales | Saprospiraceae | Portibacter |
| 2 | 0,36 | 0,0387 | * | Bacteroidota | Bacteroidia | Chitinophagales | Saprospiraceae | Portibacter |
| 2 | 0,35 | 0,0398 | * | Bacteroidota | Bacteroidia | Flavobacteriales | Crocinitomicaceae | Crocinitomix |
| 2 | 0,40 | 0,0167 | * | Bacteroidota | Bacteroidia | Flavobacteriales | Crocinitomicaceae | Lishizhenia |
| 2 | 0,36 | 0,0158 | * | Bacteroidota | Bacteroidia | Flavobacteriales | Cryomorphaceae | Unclassified |
| 2 | 0,45 | 0,0405 | * | Bacteroidota | Bacteroidia | Flavobacteriales | Flavobacteriaceae | Aquibacter |
| 2 | 0,49 | 0,0171 | * | Bacteroidota | Bacteroidia | Flavobacteriales | Flavobacteriaceae | Aquibacter |
| 2 | 0,34 | 0,0493 | * | Bacteroidota | Bacteroidia | Flavobacteriales | Flavobacteriaceae | Gramella |
| 2 | 0,40 | 0,0400 | * | Bacteroidota | Bacteroidia | Flavobacteriales | Flavobacteriaceae | Unclassified_Flavobacteriaceae |
| 2 | 0,40 | 0,0139 | * | Bacteroidota | Bacteroidia | Flavobacteriales | Flavobacteriaceae | Unclassified_Flavobacteriaceae |
| 2 | 0,40 | 0,0195 | * | Bacteroidota | Bacteroidia | Flavobacteriales | Flavobacteriaceae | Nonlabens |
| 2 | 0,32 | 0,0229 | * | Bacteroidota | Bacteroidia | Flavobacteriales | Flavobacteriaceae | Salegentibacter |
| 2 | 0,35 | 0,0220 | * | Bacteroidota | Bacteroidia | Flavobacteriales | Flavobacteriaceae | Salinimicrobium |
| 2 | 0,23 | 0,0162 | * | Bacteroidota | Bacteroidia | Flavobacteriales | Flavobacteriaceae | Ulvibacter |
| 2 | 0,30 | 0,0405 | * | Bacteroidota | Bacteroidia | Flavobacteriales | Unclassified_Flavobacteriales | Unclassified_Flavobacteriales |
| 2 | 0,45 | 0,0075 | ** | Bacteroidota | Bacteroidia | Flavobacteriales | Weeksellaceae | Empedobacter |
| 2 | 0,47 | 0,0027 | ** | Bacteroidota | Bacteroidia | Flavobacteriales | Weeksellaceae | Empedobacter |
| 2 | 0,40 | 0,0059 | ** | Bacteroidota | Rhodothermia | Balneolales | Balneolaceae | Unclassified_Balneolaceae |
| 2 | 0,40 | 0,0491 | * | Bacteroidota | Rhodothermia | Rhodothermales | Rhodothermaceae | Unclassified_Rhodothermaceae |
| 2 | 0,39 | 0,0120 | * | Bacteroidota | Rhodothermia | Rhodothermales | Rhodothermaceae | Rubrivirga |
| 2 | 0,40 | 0,0414 | * | Bdellovibrionota | Bdellovibrionia | Bacteriovoracales | Bacteriovoracaceae | Halobacteriovorax |
| 2 | 0,46 | 0,0165 | * | Campylobacterota | Campylobacteria | Campylobacterales | Arcobacteraceae | Malaciobacter |
| 2 | 0,28 | 0,0414 | * | Chloroflexi | Anaerolineae | Ardenticatenales | Ardenticateceae | Unclassified_Ardenticateceae |
| 2 | 0,28 | 0,0490 | * | Desulfobacterota | Desulfuromonadia | Bradymonadales | Unclassified_Bradymonadales | Unclassified_Bradymonadales |
| 2 | 0,59 | 0,0004 | *** | Firmicutes | Bacilli | Exiguobacterales | Exiguobacteraceae | Exiguobacterium |
| 2 | 0,34 | 0,0406 | * | Firmicutes | Clostridia | Peptostreptococcales-Tissierellales | Family XI | Aerococcus |
| 2 | 0,29 | 0,0143 | * | Firmicutes | Clostridia | Peptostreptococcales-Tissierellales | Family XI | Finegoldia |
| 2 | 0,32 | 0,0155 | * | Firmicutes | Clostridia | Peptostreptococcales-Tissierellales | Family XI | Finegoldia |
| 2 | 0,44 | 0,0439 | * | Firmicutes | Clostridia | Peptostreptococcales-Tissierellales | Unclassified_Tissierellales | Unclassified_Tissierellales |
| 2 | 0,38 | 0,0431 | * | Marinimicrobia (SAR406 clade) | Unclassified_Marinimicrobia | Unclassified_Marinimicrobia | Unclassified_Marinimicrobia | Unclassified_Marinimicrobia (SAR406 clade) |
| 2 | 0,42 | 0,0243 | * | Patescibacteria | Gracilibacteria | Absconditabacteriales (SR1) | Unclassified_Absconditabacteriales (SR1) | Unclassified_Absconditabacteriales (SR1) |
| 2 | 0,25 | 0,0398 | * | Proteobacteria | Alphaproteobacteria | Caulobacterales | HyphomoUnclassifieddaceae | Hellea |
| 2 | 0,35 | 0,0493 | * | Proteobacteria | Alphaproteobacteria | Caulobacterales | Parvularculaceae | Parvularcula |
| 2 | 0,35 | 0,0348 | * | Proteobacteria | Alphaproteobacteria | Micavibrionales | Micavibrioceae | Unclassified_Micavibrioceae |
| 2 | 0,34 | 0,0050 | ** | Proteobacteria | Alphaproteobacteria | Parvibaculales | PS1 clade | Unclassified_PS1clade |
| 2 | 0,43 | 0,0273 | * | Proteobacteria | Alphaproteobacteria | Rhizobiales | Unclassified_Rhizobiales | Unclassified_Rhizobiales |
| 2 | 0,24 | 0,0027 | ** | Proteobacteria | Alphaproteobacteria | Rhizobiales | Rhizobiaceae | Hoeflea |
| 2 | 0,23 | 0,0346 | * | Proteobacteria | Alphaproteobacteria | Rhizobiales | Rhizobiaceae | Unclassified_Rhizobiaceae |
| 2 | 0,31 | 0,0396 | * | Proteobacteria | Alphaproteobacteria | Rhizobiales | Rhizobiaceae | Roseitalea |
| 2 | 0,39 | 0,0466 | * | Proteobacteria | Alphaproteobacteria | Rhodobacterales | Rhodobacteraceae | Amylibacter |
| 2 | 0,30 | 0,0090 | ** | Proteobacteria | Alphaproteobacteria | Rhodobacterales | Rhodobacteraceae | Ascidiaceihabitans |
| 2 | 0,29 | 0,0248 | * | Proteobacteria | Alphaproteobacteria | Rhodobacterales | Rhodobacteraceae | Unclassified_Rhodobacteraceae |
| 2 | 0,34 | 0,0159 | * | Proteobacteria | Alphaproteobacteria | Rhodobacterales | Rhodobacteraceae | Unclassified_Rhodobacteraceae |
| 2 | 0,35 | 0,0391 | * | Proteobacteria | Alphaproteobacteria | Rhodobacterales | Rhodobacteraceae | Unclassified_Rhodobacteraceae |
| 2 | 0,36 | 0,0092 | ** | Proteobacteria | Alphaproteobacteria | Rhodobacterales | Rhodobacteraceae | Unclassified_Rhodobacteraceae |
| 2 | 0,42 | 0,0182 | * | Proteobacteria | Alphaproteobacteria | Rhodobacterales | Rhodobacteraceae | Unclassified_Rhodobacteraceae |
| 2 | 0,46 | 0,0058 | ** | Proteobacteria | Alphaproteobacteria | Rhodobacterales | Rhodobacteraceae | Unclassified_Rhodobacteraceae |
| 2 | 0,57 | 0,0029 | ** | Proteobacteria | Alphaproteobacteria | Rhodobacterales | Rhodobacteraceae | Unclassified_Rhodobacteraceae |
| 2 | 0,33 | 0,0319 | * | Proteobacteria | Alphaproteobacteria | Rhodobacterales | Rhodobacteraceae | Palleronia-Pseudomaribius |
| 2 | 0,33 | 0,0150 | * | Proteobacteria | Alphaproteobacteria | Rhodobacterales | Rhodobacteraceae | Roseovarius |
| 2 | 0,30 | 0,0495 | * | Proteobacteria | Alphaproteobacteria | Rhodobacterales | Rhodobacteraceae | Tateyamaria |
| 2 | 0,37 | 0,0414 | * | Proteobacteria | Alphaproteobacteria | Rickettsiales | Unclassified_Rickettsiales | Unclassified_Rickettsiales |
| 2 | 0,35 | 0,0484 | * | Proteobacteria | Alphaproteobacteria | SAR11 clade | Clade II | Unclassified_Clade II |
| 2 | 0,36 | 0,0067 | ** | Proteobacteria | Alphaproteobacteria | Sphingomonadales | Sphingomonadaceae | Altererythrobacter |
| 2 | 0,36 | 0,0249 | * | Proteobacteria | Alphaproteobacteria | Sphingomonadales | Sphingomonadaceae | Altererythrobacter |
| 2 | 0,44 | 0,0177 | * | Proteobacteria | Alphaproteobacteria | Sphingomonadales | Sphingomonadaceae | Altererythrobacter |
| 2 | 0,36 | 0,0308 | * | Proteobacteria | Alphaproteobacteria | Sphingomonadales | Sphingomonadaceae | Erythrobacter |
| 2 | 0,36 | 0,0255 | * | Proteobacteria | Alphaproteobacteria | Sphingomonadales | Sphingomonadaceae | Erythrobacter |
| 2 | 0,36 | 0,0358 | * | Proteobacteria | Gammaproteobacteria | Arenicellales | Arenicellaceae | Arenicella |
| 2 | 0,40 | 0,0161 | * | Proteobacteria | Gammaproteobacteria | Burkholderiales | ComamoUnclassifieddaceae | Delftia |
| 2 | 0,25 | 0,0392 | * | Proteobacteria | Gammaproteobacteria | Ectothiorhodospirales | Ectothiorhodospiraceae | Unclassified |
| 2 | 0,47 | 0,0002 | *** | Proteobacteria | Gammaproteobacteria | Enterobacterales | Aeromonadaceae | Aeromonas |
| 2 | 0,32 | 0,0376 | * | Proteobacteria | Gammaproteobacteria | Enterobacterales | Colwelliaceae | Thalassotalea |
| 2 | 0,28 | 0,0405 | * | Proteobacteria | Gammaproteobacteria | Enterobacterales | Kangiellaceae | Kangiella |
| 2 | 0,33 | 0,0396 | * | Proteobacteria | Gammaproteobacteria | Enterobacterales | Shewanellaceae | Shewanella |
| 2 | 0,34 | 0,0158 | * | Proteobacteria | Gammaproteobacteria | Enterobacterales | Shewanellaceae | Shewanella |
| 2 | 0,44 | 0,0398 | * | Proteobacteria | Gammaproteobacteria | Enterobacterales | Vibrionaceae | Unclassified_Vibrioceae |
| 2 | 0,48 | 0,0064 | ** | Proteobacteria | Gammaproteobacteria | Enterobacterales | Vibrionaceae | Unclassified_Vibrioceae |
| 2 | 0,32 | 0,0353 | * | Proteobacteria | Gammaproteobacteria | Enterobacterales | Vibrionaceae | Vibrio |
| 2 | 0,34 | 0,0171 | * | Proteobacteria | Gammaproteobacteria | Enterobacterales | Vibrionaceae | Vibrio |
| 2 | 0,35 | 0,0088 | ** | Proteobacteria | Gammaproteobacteria | Enterobacterales | Vibrionaceae | Vibrio |
| 2 | 0,39 | 0,0203 | * | Proteobacteria | Gammaproteobacteria | Enterobacterales | Vibrionaceae | Vibrio |
| 2 | 0,44 | 0,0387 | * | Proteobacteria | Gammaproteobacteria | Enterobacterales | Vibrionaceae | Vibrio |
| 2 | 0,36 | 0,0432 | * | Proteobacteria | Gammaproteobacteria | Unclassified_gammaproteo | Unclassified_gammaproteo | Unclassified_gammaproteo |
| 2 | 0,41 | 0,0414 | * | Proteobacteria | Gammaproteobacteria | Unclassified_gammaproteo | Unclassified_gammaproteo | Unclassified_gammaproteo |
| 2 | 0,41 | 0,0431 | * | Proteobacteria | Gammaproteobacteria | Unclassified_gammaproteo | Unclassified_gammaproteo | Unclassified_gammaproteo |
| 2 | 0,50 | 0,0150 | * | Proteobacteria | Gammaproteobacteria | Unclassified_gammaproteo | Unclassified_gammaproteo | Unclassified_gammaproteo |
| 2 | 0,38 | 0,0400 | * | Proteobacteria | Gammaproteobacteria | Pseudomonadales | Cellvibrioceae | Unclassified |
| 2 | 0,36 | 0,0402 | * | Proteobacteria | Gammaproteobacteria | Pseudomonadales | Cellvibrioceae | Pseudoteredinibacter |
| 2 | 0,44 | 0,0030 | ** | Proteobacteria | Gammaproteobacteria | Pseudomonadales | Marinobacteraceae | Marinobacter |
| 2 | 0,37 | 0,0254 | * | Proteobacteria | Gammaproteobacteria | Pseudomonadales | Nitrincolaceae | Unclassified |
| 2 | 0,37 | 0,0447 | * | Proteobacteria | Gammaproteobacteria | Pseudomonadales | Nitrincolaceae | Profundimonas |
| 2 | 0,41 | 0,0300 | * | Proteobacteria | Gammaproteobacteria | Pseudomonadales | Pseudomonadaceae | Pseudomonas |
| 2 | 0,48 | 0,0015 | ** | Proteobacteria | Gammaproteobacteria | Pseudomonadales | Pseudomonadaceae | Pseudomonas |
| 2 | 0,37 | 0,0143 | * | Proteobacteria | Gammaproteobacteria | Pseudomonadales | Saccharospirillaceae | Thalassolituus |
| 2 | 0,37 | 0,0154 | * | Proteobacteria | Gammaproteobacteria | Pseudomonadales | SAR86 clade | Unclassified_SAR86 clade |
| 2 | 0,23 | 0,0382 | * | Proteobacteria | Gammaproteobacteria | Pseudomonadales | Spongiibacteraceae | BD1-7 clade |
| 2 | 0,34 | 0,0255 | * | Proteobacteria | Gammaproteobacteria | Pseudomonadales | Spongiibacteraceae | BD1-7 clade |
| 2 | 0,32 | 0,0456 | * | Proteobacteria | Gammaproteobacteria | Salinisphaerales | Salinisphaeraceae | Salinisphaera |
| 2 | 0,33 | 0,0203 | * | Proteobacteria | Gammaproteobacteria | Salinisphaerales | Salinisphaeraceae | Salinisphaera |
| 2 | 0,36 | 0,0394 | * | Proteobacteria | Gammaproteobacteria | Salinisphaerales | Salinisphaeraceae | Salinisphaera |
| 2 | 0,41 | 0,0070 | ** | Proteobacteria | Gammaproteobacteria | Salinisphaerales | Salinisphaeraceae | Salinisphaera |
| 2 | 0,41 | 0,0171 | * | Proteobacteria | Gammaproteobacteria | Thiotrichales | Thiotrichaceae | Thiothrix |
| 2 | 0,33 | 0,0414 | * | Proteobacteria | Gammaproteobacteria | UBA10353 marine group | Unclassified_UBA10353 marine group | Unclassified_UBA10353 marine group |
| 2 | 0,44 | 0,0147 | * | Verrucomicrobiota | Verrucomicrobiae | Verrucomicrobiales | Rubritaleaceae | Persicirhabdus |
| 2 | 0,34 | 0,0435 | * | Verrucomicrobiota | Verrucomicrobiae | Verrucomicrobiales | Rubritaleaceae | Rubritalea |
| 2 | 0,39 | 0,0152 | * | Verrucomicrobiota | Verrucomicrobiae | Verrucomicrobiales | Rubritaleaceae | Rubritalea |

**Table S2.** **DESeq2 Phylum results summary table**.

|  | **baseMean** | **log2FoldChange** | **lfcSE** | **stat** | **pvalue** | **padj** |
| --- | --- | --- | --- | --- | --- | --- |
| **Chloroflexi** | 10.24 | -1.57 | 0.54 | -2.91 | 3.60E-03 | 1.44E-02 |
| **Verrucomicrobiota** | 338.70 | -1.46 | 0.33 | -4.39 | 1.15E-05 | 1.15E-04 |
| **Bacteroidota** | 5490.39 | -0.86 | 0.27 | -3.15 | 1.63E-03 | 1.09E-02 |
| **Actinobacteriota** | 263.56 | -0.70 | 0.24 | -2.99 | 2.80E-03 | 1.40E-02 |
| **Firmicutes** | 7820.48 | 1.96 | 0.30 | 6.43 | 1.28E-10 | 2.56E-09 |

**Figure S1. Track of reads remaining after each preprocessing step.** On average over 68% of the raw sequencing reads were retained after filtering, denoising, and removing chimeric sequences.


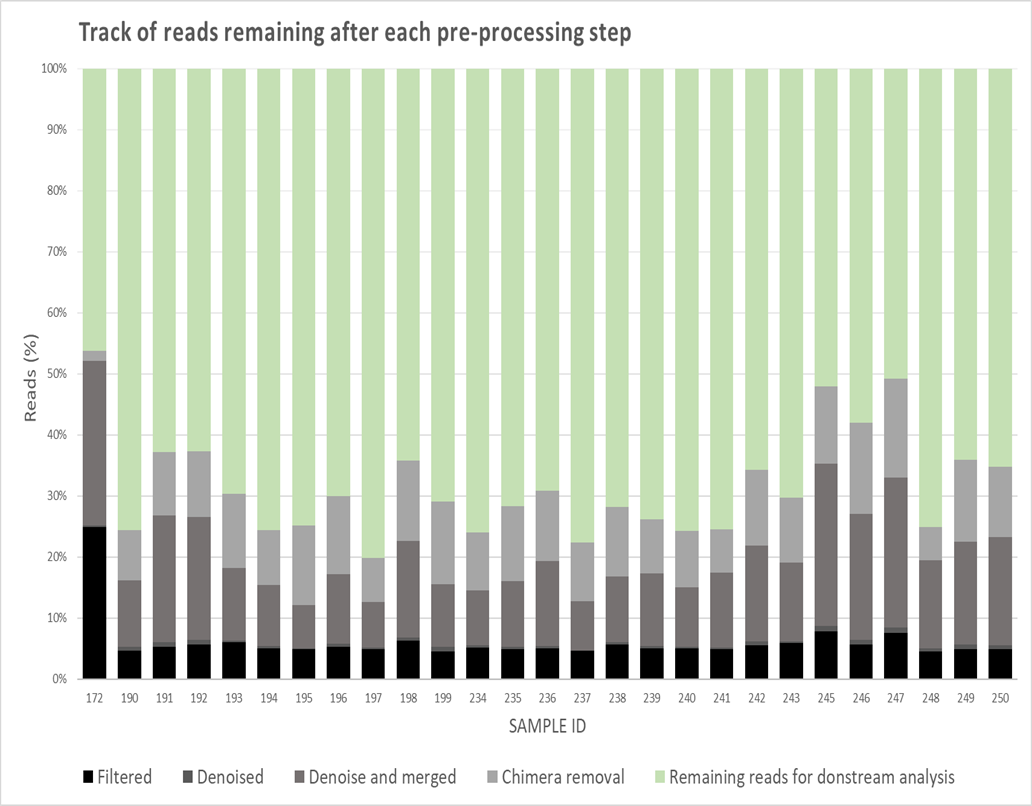


**Figure S2. Boxplot representing alpha diversity variation (Observed ASVs, Chao 1 index and Shannon diversity index) in skin and mantle cavity walls (MCW) between *O. vulgaris* females (Purple) and males (dark green).** No significant differences in skin or mantle mucosa microbial richness and diversity between Octopus females and males were detected in this study (p >0.05)


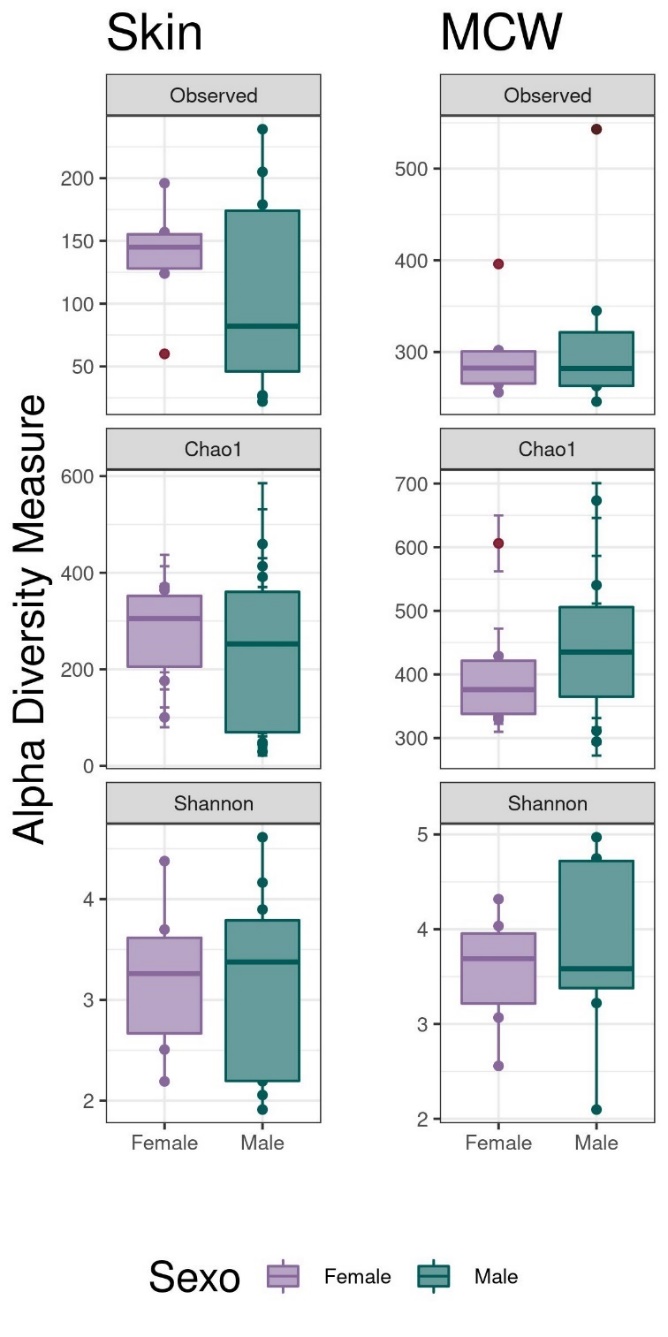


**Figure S3. DESeq2 Results Summary Dot Plot**. Each point of this plot represents a differentially abundant genus according to the DESeq results. The X-axis represents each genus for which a significant difference between sexes was found [padj (FDR) < 0.1] ordered alphabetically. The Y-axis represents the effect size (log2FC), and the dot color and size represent the significance (FDR). The dots above the horizontal line (0) were significantly higher in Females than males, and those below were significantly higher in males. Those genera that show significantly different abundances and were identified as female or male indicator species according to the indicator species analysis are highlighted with a purple or green circle, respectively, below genus name in the X-axis.

**
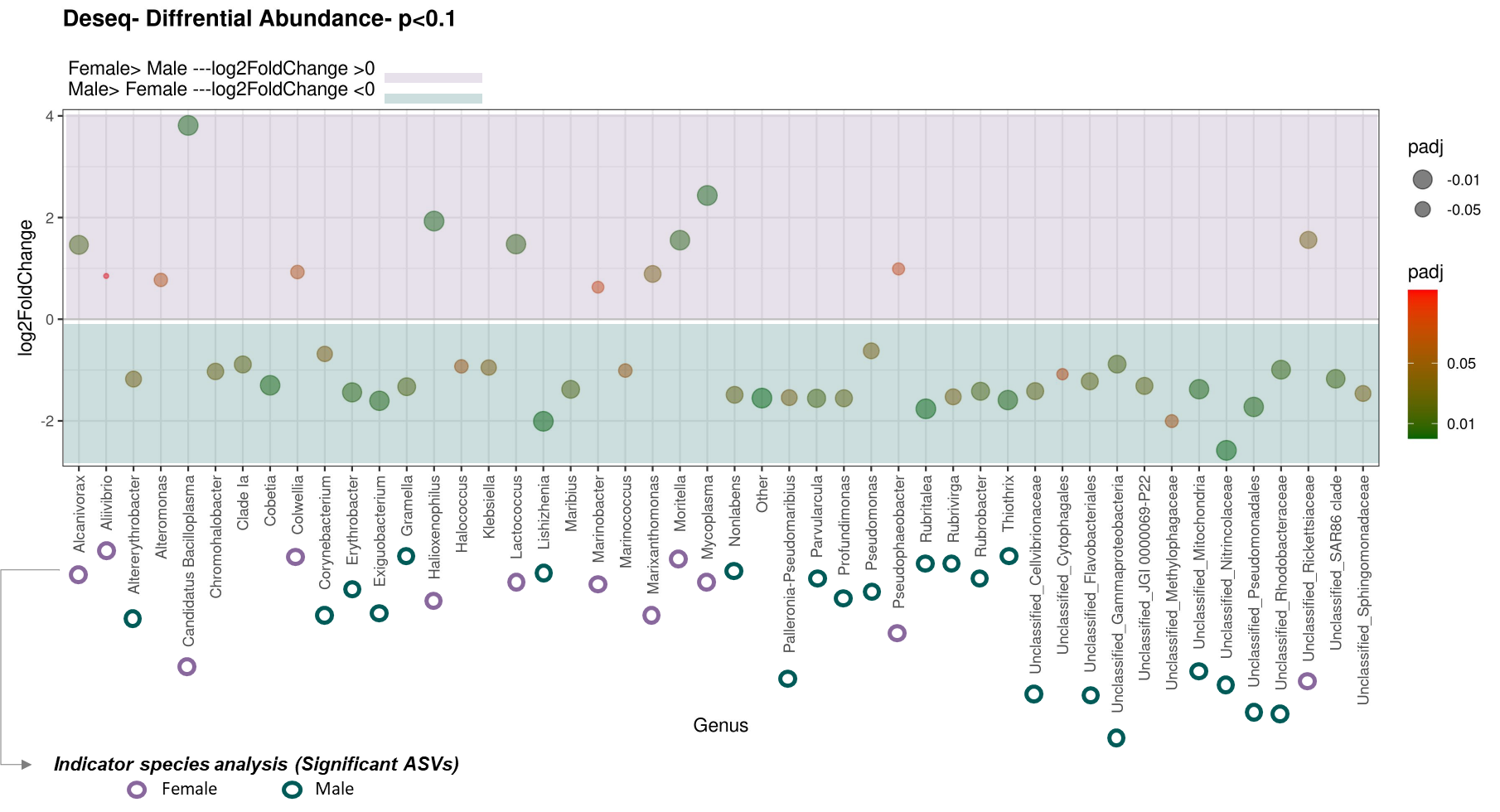
**

**Figure S4. DESeq2 Results Summary Dot Plot**. Each point of this plot represents a differentially abundant order according to the DESeq results. The X-axis represents each genus for which a significant difference between sexes was found [padj (FDR) < 0.1] ordered alphabetically. The Y-axis represents the effect size (log2FC), and the dot color and size represent the significance (FDR). The dots above the horizontal line (0) were significantly higher in Females than males, and those below were significantly higher in males.


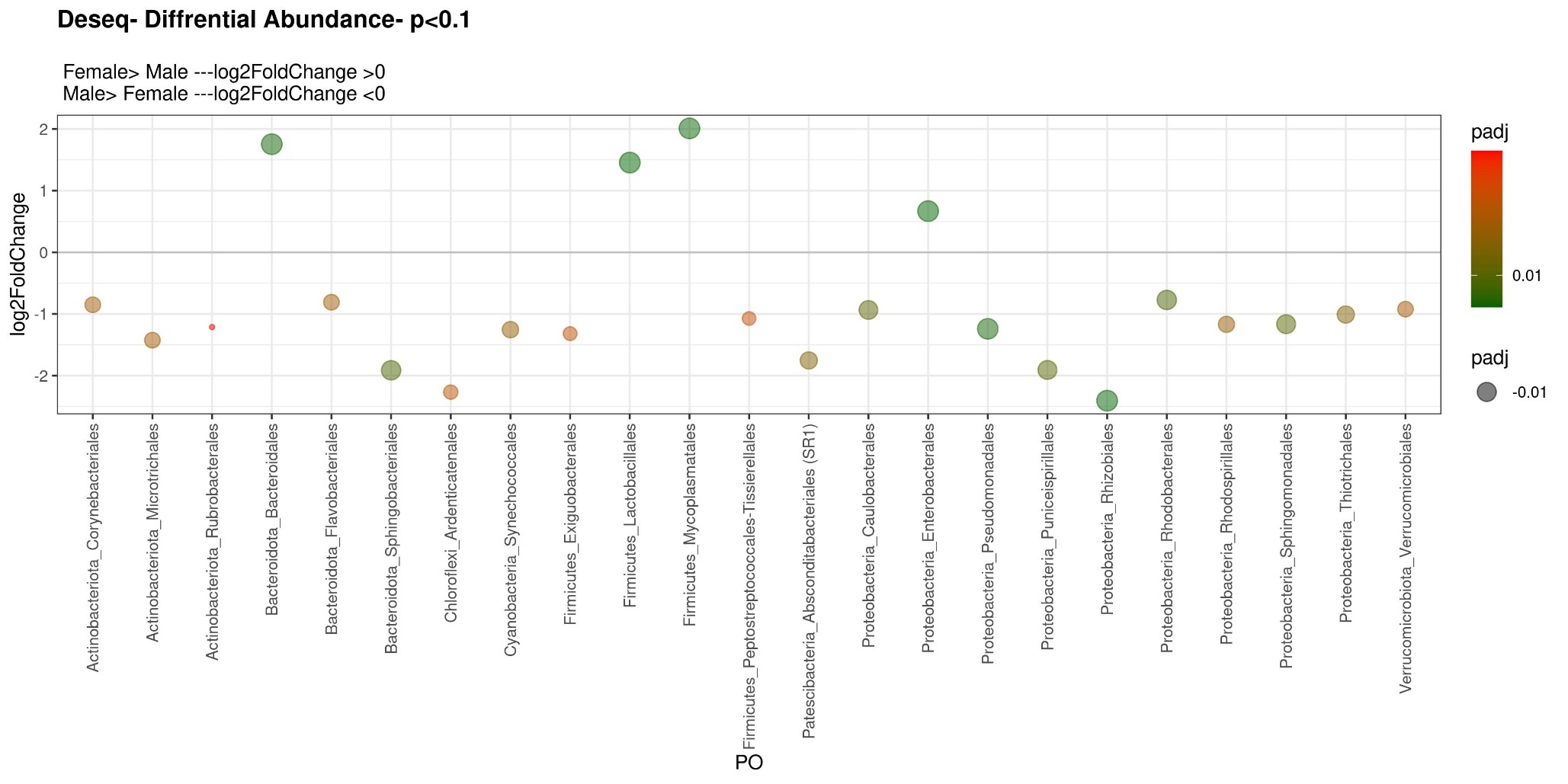

Supplement: Supplementary file 1 [file Data_Sheet_1.docx]
